# Sir Almroth Wright on Woman Suffrage

**Published:** 1913-10-25

**Authors:** 


### The Hospital

The Workers' Newspaper of Administrative Medicine and Institutional Life, Administration, National Insurance and Health.

No. 1426, Vol. LV. SATURDAY, OCTOBER 25, 1913. PRICE ONE PENNY.

### SIR ALMROTH WRIGHT ON WOMAN SUFFRAGE.

It does not seem to be perceived what near interest medical institutional workers have in the question of woman suffrage. Lay opinion, influenced by the innate (or as good as innate) aptitude of women for nursing, and by their comparative inexpensiveness, is always inclined to concede feminine claims to the management of the care of the sick. Increase the political importance of women and obviously such claims are likely to increase too, both in number and in extent. In that event lay opinion and medical opinion would probably fall out. The former would remain complaisant, but the latter might not. Few more heartily than medical men greet the matron, the ward sister, the district nurse; but what would they say to a feminine quorum on the hospital committee or—to descend momentarily into rhetoric—a crankish head of the Board of Trade run by a petticoat faction? It is unnecessary to name further eventualities of the kind, nor do we express any opinion on them. All we are concerned to show is that this way there lie ahead sundry risks of friction.

Whatever their prepossessions, however, all institutional workers might well spend half-a-crown and a couple of evenings over the essay* of Sir Almroth Wright, who by consent of all is one of our few medical men of fine intellectuality. Let none be scared by the title. In one way the book is quite (although she would never get through it) for the *jeune fille*. Indeed, the author thus misses a point in his indictment of extremists, among whom are certain unfortunate souls moved by unnatural hatred of man and unnatural love for their own sex; a fact recognised quite frankly now on the Continent, and which is due to be recognised in this country in a decade or two. We will not follow closely the arguments Sir Almroth uses, because it is always unpleasant to generalise unfavourably on the capabilities (any but a favourable verdict is only possible on *some* of the capabilities) of women. And with one of his objections we venture to disagree. He sees impossibility in men and women working side by side. This comes queerly from an actual hospital officer. In nearly every medical institution in the world the closest co-operation between males and females is a matter of course, and as human arrangements go has turned out very well. Surely every fairly normal man learns, after a certain age, to restrain his appetite for love-making when he has his daily task to do just as, at a still earlier epoch, he learnt to be able to sit in the dining room between meals without pecking at the food on the sideboard. The few exceptional individuals who lack ordinary self-control soon become known and suffer accordingly. This obvious point, which springs to the mind of the medical critic, seems to have been missed by many reviewers who, to judge by the tone of their remarks, would have been glad enough to make it. Mr. Shaw—again, by no very extraordinary chance, to be found setting a medical man to rights—can apparently think of no better refutation than to argue that since the reason of man is notoriously "valet to the emotions," it is no wonder that in women it should be correspondingly the lady's maid. But there is here one of that gentleman's characteristically huge and vital, characteristically tranquil, omissions. In woman, as Mr. Havelock Ellis could tell him, the emotions trespass upon the will and the intellect much more than they do in men. This is why women make such good nurses—and why, or so some people of experience say (since every excellence has to be compensated) they might not be such a success in other capacities.

Further than this it is not for the medical writer to go; even already he has a sense of balancing on a knife-edge, feels a shaking at the midriff, half laughter, half appreciation, just as Charles Lamb and his fellow journalists did when making their flourishes on the topic of a feminine fashion (the time repeats itself) of flesh-coloured hose. However, whatever suffrage opinions any cultured reader may hold, he will find refreshing the honesty with which the eminent pathologist states his side

* *The Unexpurgated Case Against Woman Suffrage.* By Sir A. Wright. London: Constable and Co. 1913.

of the matter. Is this quality of candour in some part the result of scientific training? Politicians make evasive, obfuscatory, truth-burking, rhetorical deliverances on the subject, sometimes, strangely enough, considerably adding to their reputations thereby. When the murder, which they seem so much afraid of, is out, is it really a murder at all? And, by the way, down at St. Mary's would they not laugh at Mr. Shaw's description of their immunisator as a nervous semi-misogynist? In the work of a clever contemporary novelist (a woman, be it said) there is a picture of a German professor of European fame to whom is introduced a young and very serious-minded Girton admirer. After the interview, in place of the expected eulogy of feminine intellect, the astonished introducer hears just these words: "A nice little, round little girl; *kolossal appetitlich*!" Let flustered pseudo-idealists think this well over and say, at long last, whether they are as shocked as they were at first. And now enough for the present of the Elian effort, with a good deal less than Elian resources, "of touching that nice brink and yet never tumbling over it, of balancing betwixt decorums and their opposites, of hovering in the confines of light and darkness, or where 'both seem either.'"

